# The Effect of a Multidisciplinary Lifestyle Intervention on Health Parameters in Children versus Adolescents with Severe Obesity

**DOI:** 10.3390/nu14091795

**Published:** 2022-04-25

**Authors:** Kelly G. H. van de Pas, Judith W. Lubrecht, Marijn L. Hesselink, Bjorn Winkens, François M. H. van Dielen, Anita C. E. Vreugdenhil

**Affiliations:** 1 Centre for Overweight Adolescent and Children’s Healthcare (COACH), Department of Paediatrics, Maastricht University Medical Centre, 6229 HX Maastricht, The Netherlands; judith.lubrecht@mumc.nl (J.W.L.); marijn.hesselink@mumc.nl (M.L.H.); a.vreugdenhil@mumc.nl (A.C.E.V.); 2 Department of Surgery, Máxima Medical Center, 5504 DB Veldhoven, The Netherlands; f.vandielen@mmc.nl; 3 School of Nutrition and Translational Research in Metabolism (NUTRIM), Maastricht University, 6229 ER Maastricht, The Netherlands; 4 Department of Methodology and Statistics, Care and Public Health Research Institute (CAPHRI), Maastricht University, 6229 ER Maastricht, The Netherlands; bjorn.winkens@maastrichtuniversity.nl

**Keywords:** multidisciplinary lifestyle intervention, children, adolescents, severe obesity

## Abstract

Lifestyle interventions are the common treatment for children and adolescents with severe obesity. The efficacy of these interventions across age groups remain unknown. Therefore, this study aimed to compare the effectiveness of a lifestyle intervention on health parameters between children and adolescents with severe obesity. A longitudinal design was carried out at the Centre for Overweight Adolescent and Children’s Healthcare (COACH) between December 2010 and June 2020. Children (2–11 years old, n = 83) and adolescents (12–18 years old, n = 77) with severe obesity received a long-term, tailored, multidisciplinary lifestyle intervention. After 1 year, 24 children (28.9%) and 33 adolescents (42.9%) dropped out of the intervention. The primary outcome was the change in body mass index (BMI) z-score after one and two years of intervention. The decrease in BMI z-score over time was significantly higher in children compared to adolescents, the mean decrease was 0.15 (0.08–0.23) versus 0.03 (−0.05–0.11) after one year and 0.25 (0.15–0.35) versus 0.06 (−0.06–0.17) after two years of intervention; *p* values for the difference between children and adolescents were 0.035 and 0.012. After two years, multiple improvements in cardio metabolic health parameters were observed, especially in children. In conclusion, during our tailored lifestyle intervention, a positive and maintained effect on health parameters was observed in children with severe obesity. Compared to children, the effect on health parameters was less pronounced in adolescents.

## 1. Introduction

Childhood obesity is a global health crisis that is recognized by the World Health Organization [1]. In 2016, 124 million children and adolescents were affected by obesity [2]. Despite the continuous efforts that are being made to reduce the prevalence of childhood obesity, there is a growing concern about the rapidly growing rates of severe obesity in children and adolescents [3,4,5,6]. In particular, the severe grade of obesity is worrisome, since children and adolescents with severe obesity have an increased cardiovascular risk compared to those with obesity [7,8]. As a consequence of this, adolescents with severe obesity have an elevated risk for the development of a fatal cardiac event later in life [9].

Multidisciplinary lifestyle intervention programs that focus on nutrition, physical activity, and behavioral change are the most frequently applied treatment options for children and adolescents with severe obesity. The Centre for Overweight Adolescent and Children’s Healthcare (COACH) offers such a lifestyle intervention program that was proven to be successful in reducing health risks in children with overweight, obesity, and severe obesity, all to a similar degree [10].

Research on multidisciplinary lifestyle interventions for children and adolescents with severe obesity is limited, with only a handful of studies examining this specific group [10,11,12,13,14,15,16,17]. A previous study reported a beneficial effect of treatment in children, but almost no effect at group level in adolescents with severe obesity [11]. A similar pattern was found by Danielsson et al., who reported that in 58% of the children with severe obesity (6–9 years old), the body mass index (BMI) z-score reduced at least 0.5 units during a three-year intervention, compared to only 2% of the adolescents (14–16 years old) [12]. Possible reasons for the limited response of adolescents to lifestyle interventions are the suggested decline in parental influence during adolescence and the reduced adherence to these interventions as age increases [18,19].

To date, there is a lack of long-term data comparing the effects of lifestyle interventions between children and adolescents with severe obesity focusing on weight loss and cardio metabolic health parameters. Therefore, this study aimed to compare the effectiveness of the COACH lifestyle intervention on weight loss and cardio metabolic health parameters between children and adolescents with severe obesity after one and two years of intervention. It was hypothesized that the COACH lifestyle intervention would result in significantly greater reductions in BMI z-score and improvements of cardio metabolic health parameters in children compared to adolescents.

## 2. Materials and Methods

### 2.1. Study Design and Population

This study was designed and conducted within COACH at the Maastricht University Medical Centre (MUMC+). A longitudinal design was used to compare the effectiveness of the COACH program between children and adolescents with severe obesity. The study was conducted according to the Declaration of Helsinki, and it was approved by the medical ethical committee of the MUMC+. It is registered at ClinicalTrial.gov as NCT02091544.

All children and adolescents with severe obesity who participated in the COACH program were eligible for inclusion in this study. Severe obesity was defined according to the International Obesity Task Force (IOTF) criteria and is comparable to a BMI ≥ 35 in adults [20]. Children were identified as participants aged 2–11 years of age at baseline, whilst adolescents were classified as those aged 12–18 years [21,22]. The age distribution is based on the transition from primary to secondary school, as it is known that this transition is a major life event and many changes occur during this period [23]. Children and adolescents with available anthropometric data after one year of intervention were included. Inclusion ran from December 2010 through to June 2020. Children and adolescents who underwent baseline assessment after June 2020 were not taken into account as one-year follow-up data were not available at the time of analysis. Participants who received a previous intervention at COACH, participants who did not receive a lifestyle intervention or received an intervention elsewhere, and participants who underwent bariatric surgery were excluded.

### 2.2. Intervention

COACH is an obesity expertise center founded in 2010, in which children with overweight or obesity and their families receive a tailored lifestyle intervention from a multidisciplinary team consisting of pediatricians, dieticians, psychologists, pedagogues, physical activity coaches, and nurses. This lifestyle intervention has been extensively described elsewhere [10]. All children and adolescents receive a baseline assessment before starting the intervention. Baseline assessment includes extensive anamnesis, physical examination, fasted blood sampling, abdominal ultrasonography, an interview with a dietician and psychologist, and questionnaires to identify underlying conditions and the presence of obesity related comorbidities. The assessment establishes an understanding of behavior and family function, and it is offered annually to children and adolescents to monitor comorbidities and weight related risk factors. The obtained information is used by the multidisciplinary team to develop an individualized, integral treatment plan. Individual guidance is offered to all families, with a focus on lifestyle changes pertaining to nutrition, food habits, physical activity, sleep, and psychosocial aspects. With regard to nutrition and food habits, the general dietary guidelines are followed and special attention is given to healthy snacks, adequate intake of fruits and dairy products, less sugar sweetened beverages, eating breakfast, adequate portion size, and shared family dinners [24]. Regarding physical activity, sleep, and social aspects the intervention focuses on limiting sedentary time, expanding physical activity, sleep hygiene, self-esteem, and emotional eating. Multiple behavioral change strategies such as motivational interviewing, goal setting, positive reinforcement, social support, and relapse prevention are employed. Individual sessions initially occur monthly, with frequency adjusted as the individual progresses through the program depending upon their individual needs. Besides the individual family guidance, the program offers possibilities to participate in sport activities and activities aimed to increase knowledge of nutrition.

### 2.3. Measurements

The primary outcome was the change in BMI z-score after one and two years of intervention between children and adolescents. Secondary outcomes were the change in cardio metabolic health parameters in and between children and adolescents after one and two years of intervention. Outcome measures were collected at baseline, and after one and two years of intervention (±four months). Data that did not fit within these time bands were excluded from analysis.

### 2.4. Anthropometric Data

Weight and height were measured barefoot. Weight was determined using digital scales (Seca), and height was measured using a digital stadiometer (De Grood Metaaltechniek). Using this information, BMI was calculated (BMI (kg/m^2^) = weight/height^2^), and BMI z-scores relative to population data from the Dutch Growth Study were obtained using a growth analyzer (Growth Analyzer VE). Children and adolescents were considered as overweight, obese, or severely obese according to the IOTF criteria [20]. Clinically significant weight loss was defined as a decrease in a BMI z-score ≥ 0.25, as improvements in body composition and cardio metabolic health parameters can be seen with this decline in the BMI z-score [25].

### 2.5. Cardio Metabolic Health Parameters

Fasting serum total cholesterol (TC), high-density lipoprotein (HDL), low-density lipoprotein (LDL), triglycerides (TG), glucose, glycated hemoglobin (HbA1c), insulin and alanine aminotransferase (ALT) concentrations were measured. Homeostatic model assessment of insulin resistance (HOMA-IR) was calculated using the formula: fasting glucose (mmol/L) * fasting insulin (mU/L)/22.5 [26]. Abnormal values of TC were determined as ≥5.2 mmol/L, for HDL as <1.0 mmol/L, for LDL as ≥3.4 mmol/L, and for TG as ≥1.5 mmol/L [27]. According to the American Diabetes Association abnormal values for fasting glucose were ≥5.6 mmol/L and for HbA1c ≥ 5.7% (39 mmol/mol) [28]. Besides this, HOMA-IR values > 2.5, and ALT values > 22 U/L (0.37 µkat/L) for females and >26 U/L (0.43 µkat/L) for males were identified as not normal [26,29].

### 2.6. Data Analysis

All data analyses were carried out using IBM SPSS Statistics for Windows (version 26, IBM Corp. Armonk, NY, USA). A two-sided *p* value ≤ 0.05 was considered statistically significant. Normality was assessed using P-P plots and histograms. Numerical data were analyzed using an independent-samples *t*-test or a Mann–Whitney U test in case of non-normal distribution and categorical data with a chi-square test to determine differences between groups at baseline. Two marginal models for repeated measures were used to assess and compare the change from baseline in the BMI z-score after one and two years of intervention in children and adolescents. Model one included Group (children/adolescents), Time (Year 1 or 2) and Group × Time as fixed factors, where an unstructured covariance structure for repeated measures was used. Next to the fixed factors included in model one, model two adjusted for potential confounders (gender, parent’s education, and ethnicity). Estimated marginal means based on restricted maximum likelihood (REML) are reported with corresponding 95% confidence intervals (CI). A likelihood-based approach for missing outcomes was applied, where variables related to missingness (logistic regression analysis) were included in the marginal model to ensure missingness at random (MAR). Due to small sample sizes, especially after one or two years of intervention, independent-samples *t*-test or Mann–Whitney U test were used to compare changes from baseline in cardio metabolic health parameters between the different age categories. Paired samples *t*-tests or Wilcoxon signed-rank tests were applied to compare changes from baseline within children and adolescents separately. In addition, abnormal values of cardio metabolic health parameters between age categories were compared using a chi-square test at each time point.

## 3. Results

### 3.1. Program Retention

**Figure 1:**
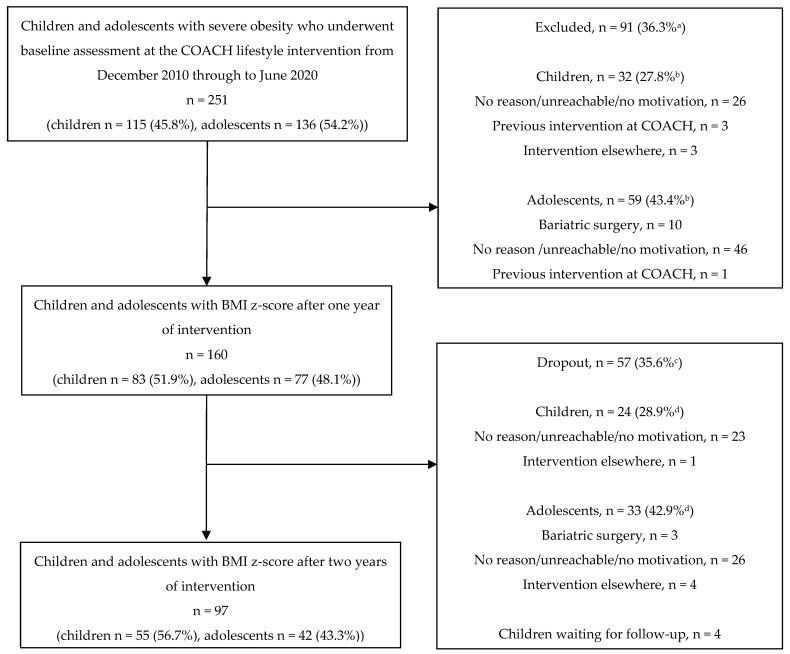
Flow diagram of the exclusion and dropout of children and adolescents with severe obesity from the COACH lifestyle intervention. ^a^ Number of excluded participants/number of participants with a baseline assessment from 2010 through 2020. ^b^ Number of children or adolescents who were excluded/number of children or adolescents with a baseline assessment from 2010 through 2020. ^c^ Dropout of the COACH lifestyle after one year of intervention/number of participants with a BMI z- score after one year of intervention. ^d^ Number of children or adolescents who dropped out of the COACH lifestyle intervention after one year of intervention/number of children or adolescents with a BMI-z-score after one year of intervention.

A total of 251 children and adolescents with severe obesity underwent baseline assessment between December 2010 and June 2020. All children and adolescents (n = 160) of whom a BMI z-score after one year of intervention was available were included in this study, reporting an exclusion rate of 36.3% (n = 91). Reasons for the missing BMI z-score after one year of intervention are mentioned in Figure 1. The included and excluded children had similar baseline characteristics, except that the included children were younger in comparison to the excluded children (11.6 ± 4.0 versus 12.9 ± 4.2 years; *p* = 0.022). In addition, significantly fewer children compared to adolescents were excluded, 27.8% (n = 32) versus 43.4% (n = 59; *p* = 0.011), respectively.

Fifty-seven children and adolescents (35.6%) included in the study dropped out of the COACH program after the first year of intervention; 24 children (28.9%) versus 33 adolescents (42.9%; *p* = 0.080). Reasons for dropout were lack of motivation, referral for bariatric surgery, or starting a lifestyle intervention elsewhere (Figure 1). A logistic regression analysis was performed to check which variables were associated with dropout of the program, but none were significant.

### 3.2. Baseline Characteristics

The baseline characteristics of the included children and adolescents were in general similar (Table 1). As expected, age, height, weight, and BMI were significantly higher in the group of adolescents. Besides this, no statistically significant differences were found regarding the cardio metabolic health parameters, except for a higher HOMA-IR and a lower HDL in the group of adolescents.

### 3.3. BMI z-Score

**Figure 2:**
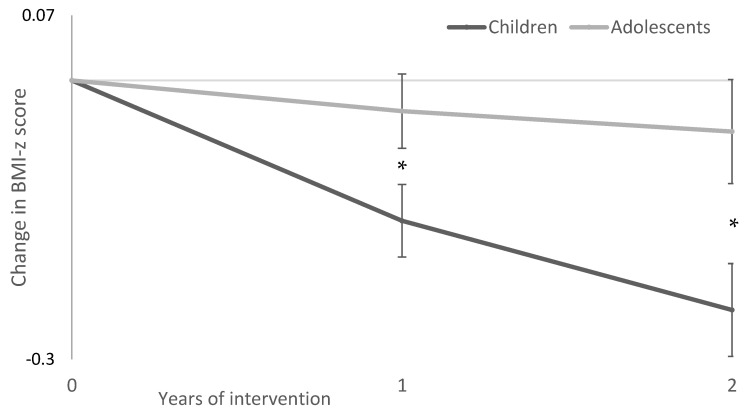
Decrease in BMI z-score after one and two years of intervention in children and adolescents determined by a marginal model for repeated measures (including group, time, and their interaction). Data presented as estimated marginal means and standard error. * *p* value ≤ 0.05, statistically different between children and adolescents.

Model one revealed that the BMI z-score of children was reduced by an additional 0.12 (0.01–0.23; *p* = 0.035) after one and an additional 0.19 (0.04–0.34; *p* = 0.012) after two years of intervention compared to the BMI z-score of adolescents (Figure 2). Children showed a significant decrease in their BMI z-score after one and two years of intervention compared to baseline, the mean decrease was 0.15 (0.08–0.23; *p* < 0.001) and 0.25 (0.15–0.35; *p* < 0.001), respectively. Adolescents showed a non-significant reduction in BMI z-score of 0.03 (−0.05–0.11; *p* = 0.417) after one year and 0.06 (−0.06–0.17; *p* = 0.316) after two years of intervention. Model two, adjusting for possible confounders (gender, parent’s education, and ethnicity), showed similar results, but only the two year difference in the change in BMI z-score between children and adolescents was significant. The BMI z-score of children was reduced by an additional 0.10 (−0.01–0.22; *p* = 0.069) and 0.18 (0.03–0.33; *p* = 0.018) after one and two years of intervention compared to adolescents.

**Figure 3:**
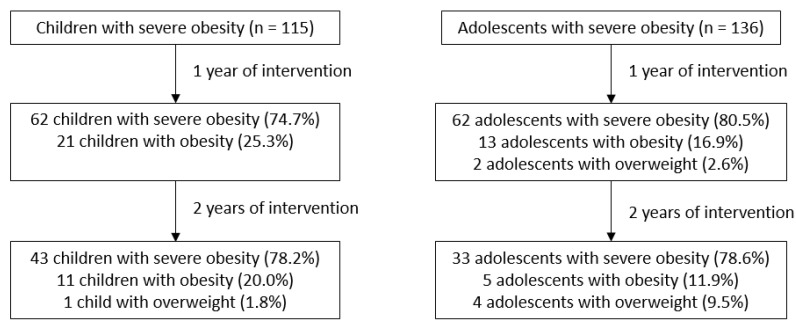
Change in IOTF criteria after one and two years of intervention presented in children and adolescents separately. Data presented as number (%). N = number, IOTF = International Obesity Task Force.

After 1 year of intervention, 21 children (25.3%) and 13 adolescents (16.9%) changed category from severe obesity to obesity, and 2 adolescents (2.6%) changed to the overweight category. After 2 years of intervention, 43 children (78.2%) were still severely obese, whereas 11 children (20.0%) switched to the obese category and 1 child (1.8%) switched to the overweight category compared to baseline. When looking at the adolescents after 2 years of intervention; 33 (78.6%) were still severely obese, 5 (11.9%) changed to the obese category and 4 (9.5%) changed to the overweight category, compared to baseline (Figure 3).

Children more often achieved a clinically significant decrease in their BMI z-score (≥0.25) compared to adolescents; 27 children (32.5%) versus 12 adolescents (15.6%; *p* = 0.013) after 1 year, and 27 children (49.1%) versus 10 adolescents (23.8%; *p* = 0.011) after 2 years of intervention. The BMI z-score of children who achieved clinically significant weight loss reduced with 0.50 ± 0.22 after 1 and 0.53 ± 0.19 after 2 years of intervention, whereas the BMI z-score of the adolescents with clinically significant weight loss decreased with 0.72 ± 0.50 after 1 and 0.81 ± 0.53 after 2 years of intervention. The majority of the children and the adolescents who did not achieve clinically significant weight loss after 1 year did not obtain this after 2 years or dropped out of the COACH program, although 12 children (21.4%) and 3 adolescents (4.6%) achieved clinically significant weight loss in the second year of intervention.

### 3.4. Cardio Metabolic Health Parameters

Regarding the changes in cardio metabolic health parameters after one and two years of intervention, no significant differences were found between the two age groups, except for the change in TC concentration after two years of intervention (Table 2). Children had a decrease in TC concentration of 0.6 ± 0.9 mmol/L, whereas adolescents had a TC decrease of 0.1 ± 0.8 mmol/L (*p* = 0.044). In children, no significant changes from baseline in cardio metabolic health parameters were observed after one year of intervention. After two years, significant decreases from baseline in TC concentration (0.6 ± 0.9 mmol/L; *p* = 0.003), LDL concentration (0.5 ± 0.6 mmol/L; *p* = 0.004) and HbA1c concentration (0.1 ± 0.2%; *p* = 0.018) were found in children. In adolescents, no significant reductions in cardio metabolic health parameters were detected after one and two years of intervention, except for HbA1c. On average, HbA1c decreased 0.2 ± 0.3% (*p* < 0.001) after 1 and 0.3 ± 0.3% (*p* = 0.006) after 2 years compared to baseline. At baseline fewer children had abnormal values of HDL and HOMA-IR compared to adolescents; 6.8% (n = 5) versus 27.0% (n = 20; *p* = 0.001) and 51.5% (n = 34) versus 78.3% (n = 54; *p* = 0.001), respectively. After one and two years of intervention, no differences between the number of children and adolescents with abnormal values of cardio metabolic health parameters were observed.

## 4. Discussion

Global authorities have recognized the need for strategies to prevent and treat severe obesity in children, as children and adolescents with severe obesity face immediate and long-term health risks [1,8,9]. This study compared the effectiveness of the COACH lifestyle intervention on health parameters between children and adolescents with severe obesity. During the long-term tailored lifestyle intervention health parameters improved in children with severe obesity, especially after two years of intervention. Compared to this younger age group, fewer improvements in health parameters were observed in adolescents with severe obesity.

The findings of the present study are in line with the findings of previously conducted research, demonstrating a larger response of lifestyle interventions in children compared to adolescents with severe obesity [11,12]. Knop et al. reported that 48.5% of the children with severe obesity reached clinically significant weight loss (defined as a BMI z-score decline of >0.25) after a one-year lifestyle intervention, whereas only 20.0% of the adolescents achieved this weight loss [11]. Our study revealed that 32.5% of the children versus 15.6% of the adolescents achieved clinically significant weight loss after 1 year of intervention, and 49.1% of the children versus 23.8% of the adolescents after 2 years of intervention. These results suggest that a selected group of adolescents respond minimally to lifestyle interventions. Therefore, future research should focus on identifying this selected group of non-responsive adolescents. For this particular group other treatment options should be sought such as enhanced lifestyle interventions or additional medical and surgical interventions.

Although not well understood, the difference in the effectiveness of lifestyle interventions between children and adolescents might be explained by, amongst others, a declined influence of parents during adolescence [18]. Previous research has shown the importance of parental involvement in childhood obesity interventions [8]. The diminished parental influence and the increasing autonomy of the adolescents may also explain the high dropout rates in our study. Secondly, adolescence is a developmental period characterized by physical and cognitive development that is accompanied by stress. Stress is associated with an increased risk of mental and cardio metabolic dysfunction and food-related coping mechanisms that might contribute to the limited effectiveness of lifestyle interventions in adolescents with severe obesity [32]. Thirdly, decreased physical activity in older compared to younger children could also partly explain the difference in the effectiveness of lifestyle interventions between children and adolescents [33].

In addition to weight loss, it is important to evaluate the effect of lifestyle interventions on cardio metabolic health parameters to assess the health risks of children and adolescents with severe obesity. Previous literature revealed the positive effects of lifestyle interventions on cardio metabolic health parameters in children and adolescents with severe obesity [10,14,15]. However, these studies did not differentiate between younger and older age groups, while it is known that the younger age group has a larger BMI z-score decrease during these interventions compared to the older age group [11,12]. Although the numbers of children and adolescents with abnormal cardio metabolic health parameters in our study were small, children had a greater decrease in TC concentration compared to adolescents after two years of intervention. Besides this, significant decreases of TC, LDL and HbA1c concentrations were found after two years of intervention in children, and in adolescents significant decreases of HbA1c were seen. In the other cardio metabolic health parameters similar trends were found although not significant, especially in the children after two years of intervention. However, the changes in cardio metabolic health parameters were small. This could be due to normal baseline values of cardio metabolic health parameters in most children and adolescents. Therefore, future studies with a larger number of participants with abnormal values of cardio metabolic health parameters are warranted to evaluate differences in the effectiveness of lifestyle interventions on cardio metabolic health parameters between children and adolescents with severe obesity.

This study has several limitations. The first limitation is the absence of a control group without a lifestyle intervention. Therefore, our study could not take into account the natural course of the weight of children and adolescents with severe obesity over time. Although, the exact natural course is unknown, it is established that youth with severe obesity are at greater risk of becoming obese in adulthood compared to youth with obesity [3]. Secondly, the two age groups may be different in terms of hereditary contributions and psychosocial factors. Unfortunately, due to the design of the study, not all these influential factors could be taken into account. Another limitation is the higher exclusion and dropout rate in the group of adolescents. Whilst this may be seen as a study limitation, it also points out an important bottleneck in the daily practice of healthcare professions, namely a lowered adherence to intervention as age increases [19]. Besides this, the available amount of data with regard to cardio metabolic health parameters after one and two years of intervention was limited. At last, body composition measurements in combination with BMI z-score and cardio metabolic health parameters would have been a more reliable indicator for weight loss and general health instead of BMI z-score and cardio metabolic health parameters alone.

## 5. Conclusions

During our tailored lifestyle intervention, a positive and maintained effect on health parameters was observed in children with severe obesity. Compared to children, the effect on health parameters was less pronounced in adolescents with severe obesity. Although a small subgroup of adolescents achieved clinically significant weight loss during the current lifestyle intervention, the majority of the adolescents were unresponsive. These results advocate starting treatment for severe obesity at an early age. Additionally, for a selected group of adolescents, enhanced lifestyle interventions possibly supplemented with medical or surgical treatment options are needed.

## Figures and Tables

**Table 1:** Baseline characteristics of the included children and adolescents with severe obesity.

	Children n = 83	Adolescents n = 77	*p* Value
Age (years, ±SD)	8.3 ± 2.4	15.2 ± 1.5	<0.001 *
Gender, no. (%)			
Female	39 (47.0)	47 (61.0)	0.075
Height (m, ±SD)	1.4 ± 0.2	1.7 ± 0.1	<0.001 *
Weight (kg, ±SD)	56.1 ± 20.5	109.4 ± 18.8	<0.001 *
BMI (kg/m^2^, ±SD)	28.6 ± 4.6	38.9 ± 5.1	<0.001 *
BMI z-score (±SD)	4.07 ± 0.55	3.96 ± 0.40	0.139
TC (mmol/L, ±SD)	4.4 ± 0.8	4.4 ± 0.9	0.872
HDL (mmol/L, ±SD)	1.2 ± 0.2	1.1 ± 0.3	0.021 *
LDL (mmol/L, ±SD)	2.6 ± 0.7	2.7 ± 0.7	0.546
TG (mmol/L, Q1, Q3)	1.1 [0.7–1.2]	1.0 [0.7–1.3]	0.729
Fasting glucose (mmol/L, ±SD)	4.3 ± 0.6	4.2 ± 0.6	0.605
HbA1c (%, ±SD)	5.3 ± 0.5	5.3 ± 0.4	0.527
HOMA-IR [Q1, Q3]	2.6 [1.4–3.7]	4.0 [2.9–5.5]	<0.001 *
ALT (U/l, Q1, Q3)	26.0 [21.0–32.0]	22.0 [16.0–36.0]	0.543
Mother’s BMI (kg/m^2^, ±SD)	31.6 ± 6.3	31.3 ± 7.0	0.843
Father’s BMI (kg/m^2^, ±SD)	29.0 ± 4.8	30.3 ± 5.1	0.158
Ethnicity, no. (%) ^a^			
Dutch	52 (62.7)	59 (77.6)	0.119
Western	8 (9.6)	4 (5.3)	
Non-Western	23 (27.7)	13 (17.1)	
Parent’s education, no. (%) ^a^			
Low	34 (42.5)	28 (37.3)	0.795
Middle	34 (42.5)	34 (45.3)	
High	12 (15.0)	13 (17.3)	

Data presented as number (%), mean ± SD or median [Q1, Q3]. * *p* value ≤ 0.05. N = number, SD = standard deviation, BMI = body mass index, TC = total cholesterol, HDL = high density lipoprotein, LDL = low density lipoprotein, TG = Triglycerides, HbA1c = glycated hemoglobin, HOMA-IR = homeostatic model assessment for insulin resistance, ALT = alanine aminotransferase. ^a^ According to the Dutch Central Agency for Statistics [30,31].

**Table 2:** Change in cardio metabolic health parameters after one and two years of intervention in and between children and adolescents.

	Children		Adolescents	
	Baseline—Year 1 Mean ± SD (n)	Baseline—Year 2 Mean ± SD (n)	Baseline—Year 1 Mean ± SD (n)	Baseline—Year 2 Mean ± SD (n)
TC (mmol/L)	−0.1 ± 0.6 (29)	−0.6 ± 0.9 (21) * ^#^	−0.1 ± 0.6 (34)	−0.1 ± 0.8 (15)
HDL (mmol/L)	0.0 ± 0.2 (29)	−0.1 ± 0.3 (21)	0.0 ± 0.2 (34)	0.0 ± 0.3 (15)
LDL (mmol/L)	−0.1 ± 0.6 (29)	−0.5 ± 0.6 (21) *	−0.1 ± 0.7 (33)	−0.1 ± 0.8 (15)
TG (mmol/L)	0.0 ± 0.4 (29)	−0.3 ± 0.6 (21)	0.1 ± 0.7 (33)	0.0 ± 0.6 (15)
Fasting glucose (mmol/L)	0.2 ± 0.6 (27)	−0.1 ± 0.7 (20)	0.2 ± 0.8 (34)	0.1 ± 0.9 (15)
HbA1c (%)	−0.1 ± 0.4 (27)	−0.1 ± 0.2 (21) *	−0.2 ± 0.3 (34) *	−0.3 ± 0.3 (15) *
HOMA-IR	0.5 ± 2.5 (23)	0.0 ± 2.5 (15)	0.5 ± 2.2 (29)	−0.3 ± 1.9 (11)
ALT (U/L)	−3.0 [−17.0–3.3] (28)	−6.5 [−27.8–0.8] (21)	0.0 [−4.0–2.0] (34)	5.0 [−13.0–22.0] (15)

Data presented as mean ± SD or median [Q1, Q3]. * *p* value ≤ 0.05, statistically different change at years 1 or 2 compared to baseline in children and adolescents separately. # *p* value ≤ 0.05, statistically different between children and adolescents. N = number, SD = standard deviation, TC = total cholesterol, HDL = high density lipoprotein, LDL = low density lipoprotein, TG = Triglycerides, HbA1c = glycated hemoglobin, HOMA-IR = homeostatic model assessment for insulin resistance, ALT = alanine aminotransferase.

## Data Availability

The data presented in this study are available on request from the corresponding author. The data are not publicly available due to ethical restrictions.
